# Supplementary material for: Hypergravity as a gravitational therapy mitigates the effects of knee osteoarthritis on the musculoskeletal system in a murine model
Source: PLoS One. 2020 Dec 9;15(12):e0243098. doi: 10.1371/journal.pone.0243098 (PMC7725345; doi:10.1371/journal.pone.0243098)
Supplement: S1 File — (DOCX) [file pone.0243098.s001.docx]

**Supplementary material, Revised version**

**“Hypergravity as a Gravitational Therapy Mitigates the Effects of Knee Osteoarthritis on the Musculoskeletal System in a murine model** “

Dechaumet B et al.

**S1 Video, the hypergravity plateform running at 2g**. General view of the centrifuge (COMAT Aérospace, Flourens, France) from the video tracking installed at the roof of the room where the centrifuge is accommodated. The centrifuge has a radius of 1.4 m had four gondolas hanging on the periphery. An acceleration of 2g (rotation speed of 29.6 rotations per minute) was fixed over the hypergravity period of 8 weeks.

<https://filesender.renater.fr/?s=download&token=2cdefbc0-b947-4e71-8d90-cc6a961be133>

Created : 13/11/2020

Expired : 11/12/2020

 11.9 Mo

**S1 Figure Inside a gondola.** Each gondola can accommodate up to four cages. All gondolas were equipped with an infra-red video surveillance system to control animals’ condition and food/water stocks.


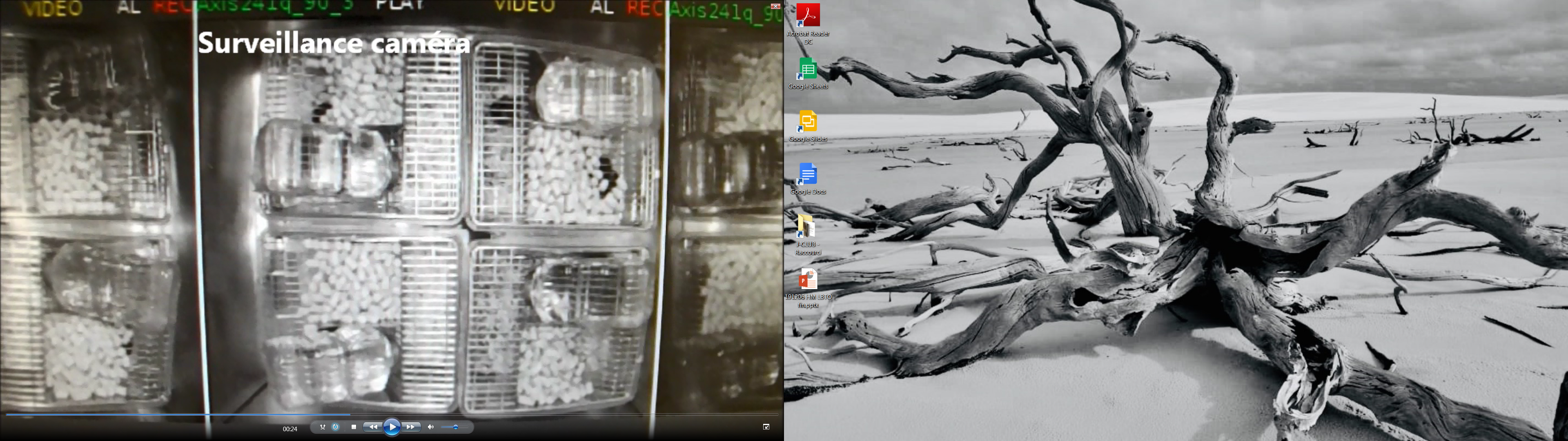


|  | Name | Condition | Forward primers | Reverse primers |
| --- | --- | --- | --- | --- |
| Housekeeping gene | **HPRT (nm_013556.2)** | 45 cycles, 60°C; | tgttggatacaggccagact | caacttgcgctcatcttag |
| Muscle genes | **VISFATIN (nm_021524.2)** | 45 cycles, 63°C | tgtctgtggtcagcgatagc | aggtggcagcaacttgtagc |
|  | **PPARγ2 (nm_001127330)** | 45 cycles, 63°C | tccgtgatggaagaccactc | ccacagactcggcactcaat |
|  | **C/EBPα (nm_007678.3)** | 40 cycles, 60°C | caagccaggactaggagatt | ccaaggcacaaggttacttc |
|  | **MYOSTATIN (nm_010834.3)** | 45 cycles, 63°C | ggccatgatcttgctgtaac | gcttggtgcacaagatgagt |
|  | **ACTIVIN RIIB (nm_007397.3)** | 45 cycles,63°C | ggctgctggctagatgactt | aaggcattggctggaagaac |
|  | **FAT/CD36 (nm_010834.3)** | 45 cycles, 63°C | gccaagctattgcgacatga | aaggcattggctggaagaac |
|  | **FNDC5** | 45 cycles, 63°C | acctggaggaggacacagaa | catactggcggcagaagaga |
| Bone genes | **OCN (nm_007541.2)** | 45 cycles, 63°C | ctctgacctcacagatgccaa | ctggtctgatagctcgtcaca |
|  | **RUNX2 (nm_009820.4)** | 45 cycles, 60°C | ccgggaatgatgagaactac | tgtctgtgccttcttggttc |
|  | **SOST (nm_024449.5)** | 45 cycles, 60°C | tcctcctgagaacaaccagac | tgtcaggaagcgggtgtagtg |
|  | **PPARγ2 (nm_001127330.1)** | 45 cycles, 63°C | tccgtgatggaagaccactc | ccacagactcggcactcaat |
|  | **C/EBPα (nm_007678.3)** | 40 cycles, 60°C | caagccaggactaggagatt | ccaaggcacaaggttacttc |
|  | **TNF-α (nm_013693.3)** | 45 cycles, 63°C | gaactggcagaagaggcact | ggccatagaactgatgagag |

**S1 Table: List of primers used for bone and muscle genes**

**S2 Table Excel file “Raw data 2g and OA Dechaumet et al. revised version”** in the following link:

<https://filesender.renater.fr/?s=download&token=1f0e42a9-7294-4647-8937-51d417aed243>

Created : 13/11/2020

Expired : 11/12/2020

70.1 ko

Legend of S2 Table “Raw data 2g and OA Dechaumet et al. revised version”

In each group Ctrl 1g, OA 1g, Ctrl 2g, OA 2g, the following parameters are reported

- OARSI, score
- Tibia, 3D metaphyseal bone nano CT
  - Blood vessel density, (/mm²)
  - BV/TV, Bone Volume/Tissue Volume (%)
  - Tb.Th, Trabecular Thickness (µm)
  - Tb.Sp, Trabecular Separation (µm)
  - Tb.N, Trabecular Number (/mm)
  - Vascular volume/Marrow Volume, (%)
- Tibia, 3D cortical bone nano CT
  - Ct.Th, Cortical Thickness (µm)
  - Ct Po, Cortical Porosity (%)
- Adipocyte density tibia metaphysis (Number per mm^2^)
- Body Weight start (g)
- Body Weight end (g)
- Tibialis wet weight left (g)
- Tibialis wet weight right (g)
- Gastrocnemuis wet weight left (g)
- Gastrocnemius wet weight right (g)
- Soleus wet weight left (g)
- Soleus wet weight right (g)
- Bone histomorphometry
  - sLS/BS, single labelled surface/Bone Surface (%)
  - dLS/BS, doubled labelled surface/Bone Surface (%)
  - MAR, Mineral Apposition Rate (µm/d)
  - BFR/BS, Bone Formation Rate/ Bone Surface (µm^3^/µm^2^/day)
  - N.Oc/B.Ar, Number of Osteoclast/Bone Area (Number/µm^2^)
  - N.Oc/ B.Pm, Number of Osteoclast/Bone Perimeter (Number/µm)
  - Oc.S/BS, Number of Osteoclast/Bone Surface (%)
  - Oc.Le, Osteoclastic Length (µm)
- Soleus
  - blood vessel /200µm²
  - % fibres with lipid droplets
  - soleus area fibers (µm²)
  - % Fibre Type 1
  - % Fibre Type 2
  - number of vessel /fibre
- Kondziela score
- Gastrocnemius, gene expression
  - Visfatin (AU)
  - Myostatin (AU)
  - Activin RIIb (AU)
  - PPAR γ (AU)
  - C-EBP α (AU)
  - FAT/CD36 (AU)
  - FNDC5 (AU)
  - Follistatine (AU)
- Serum
  - TNF alpha (pg/ml)
  - Visfatin (ng/ml)
  - Corticosterone (ng/ml)
  - Irisin (pg/ml)
- Bone, gene expression
  - SOST (AU)
  - OCN, osteocalcin (AU)
  - Runx2 (AU)
  - PPAR γ (AU)
  - C-EBP α (AU)
  - TNF α (AU)
  - FNDC5 (AU)
- Tibialis
  - Area Fiber (µm^2^)
  - Vessel density (Vessel Number/200 µm^2^)
  - Vessel Number (Number/fiber)
  - Fibers with lipid droplets (%)

**S3 Table. Average distance (in meter) travelled in the actimetry wheel / week**. Registration were made in 3 extra mice in 2g Ctrl and 2g OA groups during week 3 to 7 of the 8-week experiment. No statistical difference (Mann-Whitney test) were seen between the two groups.

|  |  |  |  |
| --- | --- | --- | --- |
|  |  | **OA 1g** | **OA 2g** |
|  | **Week 3** | 3143.15 ± 1927.09 | 2141.95 ± 674.94 |
|  | **Week 4** | 2111.86 ± 697.74 | 2405 ± 792.72 |
|  | **Week 5** | 2168.74 ± 470.1 | 2880.12 ± 1344.34 |
|  | **Week 6** | 1305.78 ± 456.42 | 2060.25 ± 1245.15 |
|  | **Week 7** | 1221.94 ± 389.23 | 1911.54 ± 699.56 |
